# Validation of a simplex PCR assay enabling reliable identification of clinically relevant *Candida* species

**DOI:** 10.1186/s12879-018-3283-6

**Published:** 2018-08-13

**Authors:** Gabor Fidler, Eva Leiter, Sandor Kocsube, Sandor Biro, Melinda Paholcsek

**Affiliations:** 1 Faculty of Medicine, Department of Human Genetics, University of Debrecen, Nagyerdei krt. 98, Debrecen, H-4032 Hungary; 2 Faculty of Science and Technology, Department of Biotechnology and Microbiology, University of Debrecen, Debrecen, Hungary; 3 Faculty of Science and Informatics, Department of Microbiology, University of Szeged, Szeged, Hungary

**Keywords:** *Candida*, High resolution melting, T_m_ calling, Species level identification, Simplex PCR

## Abstract

**Background:**

Fungal bloodstream infections (BSI) may be serious and are associated with drastic rise in mortality and health care costs. *Candida* spp. are the predominant etiological agent of fungal sepsis. The prompt and species-level identification of *Candida* may influence patient outcome and survival. The aim of this study was to develop and evaluate the CanTub-simplex PCR assay coupled with T_m_ calling and subsequent high resolution melting (HRM) analysis to barcode seven clinically relevant *Candida* species.

**Methods:**

Efficiency, coefficient of correlation and the limit of reliable detection were estimated on purified *Candida* EDTA-whole blood (WB) reference panels seeded with *Candida albicans, Candida glabrata, Candida parapsilosis, Candida tropicalis, Candida krusei, Candida guilliermondii, Candida dubliniensis* cells in a 6-log range. Discriminatory power was measured on EDTA-WB clinical panels on three different PCR platforms; LightCycler®96, LightCycler® Nano, LightCycler® 2.0. Inter- and intra assay consistencies were also calculated.

**Results:**

The limit of reliable detection proved to be 0.2–2 genomic equivalent and the method was reliable on broad concentration ranges (10^6^–10 CFU) providing distinctive melting peaks and characteristic HRM curves. The diagnostic accuracy of the discrimination proved to be the best on Roche LightCycler®2.0 platform. Repeatability was tested and proved to be % C.V.: 0.14 ± 0.06 on reference- and % C.V.: 0.14 ± 0.02 on clinical-plates accounting for a very high accuracy. Reproducibility was % C.V.: 0.11 between reference- and % C.V.: 0.12between clinical-panels which is highly acceptable.

**Conclusion:**

Our assay demonstrates recent advances on T_m_ calling and HRM analysis for the molecular identification of relevant *Candida* species. This unique, simplex PCR assay may be capable to outperform conventional phenotypic methods by reducing time and providing accurate and reliable results directly from blood (2 h) or from whole blood culture bottles (12–24 h).

**Electronic supplementary material:**

The online version of this article (10.1186/s12879-018-3283-6) contains supplementary material, which is available to authorized users.

## Background

Fungal bloodstream infections may be serious and are associated with drastic rise in mortality and health care costs [1, 2]. Currently, with the gradually growing number of immunocompromised patients, we face an appreciable shift in the spectrum of fungal infections [3, 4]. The genus *Candida* corresponds to the most important cause of opportunistic mycoses in the world [5, 6]. Despite the antifungal therapy nosocomial *Candida* infections constitute a public health problem contributing to prolonged hospitalization time, generating enormous excess of costs for patient treatment, and a high mortality (25–60%) especially when complicated with septic shock [7, 8].

*Candida* spp. account for 70–80% of invasive fungal BSIs representing the third most frequent (all over 8–10%) cause of all BSIs in the intensive care unit (ICU) [9–13]. Nearly 95% of invasive candidemias are caused by *Candida albicans*, *Candida glabrata*, *Candida parapsilosis*, *Candida tropicalis* and *Candida krusei* [14–16]. The remaining 5% are caused by 10–12 species, such as *C. guilliermondii*, *C. dubliniensis* provoking superinfections due to their low sensitivity to broad-spectrum antifungals [10, 17]. The incidence of infections due to non-*albicans* species is continuously growing [13, 17]. *Candida* BSIs do not present with species specific clinical manifestations or laboratory abnormalities, thus the clinical course of the disease and the outcome are comparable in patients with sepsis caused by *C. albicans* and non-*albicans* species [12, 18].

Timely diagnosis of sepsis due to candidemia is essential for effective therapy, while delays of more than 12 h in the administration of antifungal drugs may substantially increase mortality [19–23].

The rapid and correct identification of *Candida* species can narrow therapy options by preventing treatment with potentially toxic antifungal agents, thus reducing costs of hospitalization and improving negative patient outcomes [24–26]. Because of the phenotypic similarities of *Candida* species, the turn-around time of traditional, culture based identification methods may take 2 to 8 days, delay adequate diagnosis and appropriate antifungal treatment. In comparison the surrogate-marker based molecular assays requiring an average of 3–4 h [27]. Nevertheless in some cases the morphological identification of clinically relevant *Candida* species is hampered by several difficulties, as is the case with the germ tube positive *C. albicans* and the potentially fluconazole-resistant, cryptic *C. dubliniensis* providing light green vs. dark green colonies on CHROM agar presenting only subtle differences in colony color [28, 29].

With regard to surrogate-marker based molecular methods, the antimannan antibody (Platelia GM-enzyme immunoassay, Bio-Rad) and 1,3-ß-D-glucan (BDG) antigen based (Fungitell assay, Associates of Cape Cod) immunological assays have good performances and may be useful in diagnosing of invasive fungal infections [30]. Nonetheless, as the panfungal BDG is a major cell wall component of *Candida* and other fungi, except *Cryptococci* and *Zygomycetes*, it not suitable for the identification even to the genus level [12].

Along with the growing number of annotated protein spectra and the development of refined and standardized methodologies of protein extraction methods, matrix assisted laser desorption/ionization time-of-flight (MALDI-TOF) mass spectrometry (MS) has emerged as a powerful technology for the prompt identification of microorganisms in clinical microbiology supporting genus-, or species-oriented treatment [31–33].

The DNA sequencing methods, targeting the 18S rRNA genes or ITS regions, can generate accurate species-level identification for many microbial isolates; however these methods are time consuming and technically demanding.

Polymerase chain reaction (PCR)-based techniques appear to be promising in terms of speed, economy, and resolution power [34]. PCR based patient follow up were shown to be capable to precede clinical signs of invasive candidiasis (IC) with the range of 1 day to 4 weeks, furthermore treatment could be initiated 3 days (range: 0–8 days) before the blood culture diagnosis was taken [35]. There have been numerous platforms and gene targets used for *Candida spp*. identification, such as those genes encoding cytochrome P450, actin and L1A1, and the highly variable internal transcribed spacer regions; ITS1 and ITS2 in combination with the relatively conserved regions of 18S, 5.8S or 28S nuclear rRNA genes [36–41].

MALDI-TOF and real-time PCR applications have high throughput, low-cost in supplies, short turnaround time. Both tools have the advantage to identify a broad-range of clinically relevant pathogenes with high accuracy at the species level Though MALDI-TOF is not suitable to detect a low amount of microorganisms directly from blood and PCR techniques usually lack standardization.

The prime aim of this study is to develop and evaluate a real-time PCR method which can identify and differentiate among seven relevant *Candida* spp. with high accuracy in a single, closed tube system, by a post-PCR T_m_ calling assay coupled with a contingent high-resolution melting (HRM) analysis. This barcoding method is a single locus based HRM system which relies on the conserved regions of *Candida* beta-tubulin genes permitting reliable and precise species-specific identification of seven reference strains and 38 clinical isolates. To our knowledge, there is no other published method which is tailored to a single domain of *Candida* beta-tubulin genes and there is no other conventionally available or presented simplex PCR assay.

## Methods

### Fungal and bacterial strains

Genomic DNA (gDNA) samples of 81 clinically relevant fungal strains; *Candida* (43), aspergilli (32), *Fusarium* (4), *Lichtemia* (1), *Rhyzopus* (1), *Scedosporium* (1) and 16 bacteria; Gram-positive (10) and Gram-negative (6) were examined. The reference strains and clinical isolates (Table 1) were maintained at the Department of Microbiology, University of Szeged and at the Department of Medical Microbiology, University of Debrecen. To preserve the viability and purity of fungal and bacterial strains they were maintained in 50% glycerol stock solution at − 80 °C and were periodically subcultured.

**Table 1:** List of the reference and clinical strains examined by CanTub-simplex PCR

	DNA serial ID.	Strain designation number	Organism	T_m_ °C
Yeasts	Reference	ATCC 10231	*Candida albicans*	78.77 ± 0.06
	1	DMC 14134	*Candida albicans*	78.60 ± 0.08
	2	DMC 3666	*Candida albicans*	78.70 ± 0.11
	3	DMC 40678	*Candida albicans*	78.43 ± 0.16
	4	SZMC 22801	*Candida albicans*	78.57 ± 0.12
	5	SZMC 22800	*Candida albicans*	78.77 ± 0.02
	6	SZMC 1523	*Candida albicans*	78.69 ± 0.05
	7	SZMC 1422	*Candida albicans*	78.67 ± 0.11
	Reference	ATCC 90030	*Candida glabrata*	81.55 ± 0.03
	8	SZMC 1353	*Candida glabrata*	81.44 ± 0.14
	9	SZMC 1362	*Candida glabrata*	81.59 ± 0.12
	10	SZMC 1370	*Candida glabrata*	81.30 ± 0.15
	11	SZMC 1374	*Candida glabrata*	81.55 ± 0.14
	Reference	ATCC 22019	*Candida parapsilosis*	80.16 ± 0.05
	12	DMC 6378	*Candida parapsilosis*	80.18 ± 0.10
	13	DMC 6403	*Candida parapsilosis*	80.26 ± 0.15
	14	DMC 16879	*Candida parapsilosis*	80.26 ± 0.02
	15	SZMC 23640	*Candida parapsilosis*	79.91 ± 0.02
	16	SZMC 8114	*Candida parapsilosis*	80.18 ± 0.06
	17	SZMC 1593	*Candida parapsilosis*	80.12 ± 0.04
	Reference	ATCC 750	*Candida tropicalis*	78.47 ± 0.11
	18	DMC 10776	*Candida tropicalis*	78.13 ± 0.16
	19	DMC 2696	*Candida tropicalis*	78.36 ± 0.02
	20	DMC 3403	*Candida tropicalis*	78.25 ± 0.14
	21	SZMC 1351	*Candida tropicalis*	78.20 ± 0.10
	22	SZMC 1364	*Candida tropicalis*	78.17 ± 0.07
	23	SZMC 1366	*Candida tropicalis*	78.33 ± 0.06
	24	SZMC 1368	*Candida tropicalis*	78.13 ± 0.11
	Reference	ATCC 6258	*Candida krusei*	79.31 ± 0.05
	25	DMC 26513	*Candida krusei*	79.27 ± 0.01
	26	DMC 23697	*Candida krusei*	79.34 ± 0.06
	27	DMC 22910	*Candida krusei*	79.39 ± 0.07
	28	SZMC 1352	*Candida krusei*	79.20 ± 0.03
	29	SZMC 1414	*Candida krusei*	79.32 ± 0.05
	30	SZMC 1447	*Candida krusei*	79.24 ± 0.12
	31	SZMC 1450	*Candida guilliermondii*	81.09 ± 0.10
	32	SZMC 0808	*Candida guilliermondii*	81.01 ± 0.06
	33	SZMC 1536	*Candida guilliermondii*	81.08 ± 0.12
	34	SZMC 1357	*Candida guilliermondii*	80.84 ± 0.11
	35	SZMC 1469	*Candida dubliniensis*	77.91 ± 0.05
	36	SZMC 1470	*Candida dubliniensis*	77.75 ± 0.01
	37	SZMC 1471	*Candida dubliniensis*	77.62 ± 0.03
	38	SZMC 1472	*Candida dubliniensis*	77.62 ± 0.08
Moulds	Reference	FGSC A1156	*Aspergillus terreus*	–
	39	SZMC 22546	*Aspergillus terreus*	–
	40	SZMC 22547	*Aspergillus terreus*	–
	41	SZMC 22548	*Aspergillus terreus*	–
	42	SZMC 22549	*Aspergillus terreus*	–
	Reference	CBS 101355/AF 293	*Aspergillus fumigatus*	–
	43	SZMC 2504	*Aspergillus fumigatus*	–
	44	SZMC 2486	*Aspergillus fumigatus*	–
	45	SZMC 22550	*Aspergillus fumigatus*	–
	46	SZMC 22551	*Aspergillus fumigatus*	–
	47	SZMC 22552	*Aspergillus fumigatus*	–
	48	SZMC 22553	*Aspergillus fumigatus*	–
	49	SZMC 22554	*Aspergillus fumigatus*	–
	Reference	CBS 117885	*Aspergillus lentulus*	–
	50	SZMC 3123	*Aspergillus lentulus*	–
	51	SZMC 20911	*Aspergillus lentulus*	–
	Reference	NRRL 11611	*Aspergillus flavus*	86.36
	52	SZMC 22583	*Aspergillus flavus*	–
	53	SZMC 22582	*Aspergillus flavus*	–
	54	SZMC 22581	*Aspergillus flavus*	86.61
	55	SZMC 22580	*Aspergillus flavus*	86.34
	56	SZMC 22578	*Aspergillus flavus*	86.39
	57	SZMC 22577	*Aspergillus flavus*	–
	58	SZMC 22575	*Aspergillus flavus*	86.38
	Reference	CBS 113.46	*Aspergillus niger*	86.57
	59	SZMC 3119	*Aspergillus niger*	86.75
	60	SZMC 3108	*Aspergillus niger*	–
	Reference	CBS 134.48	*Aspergillus tubingensis*	–
	61	SZMC 3127	*Aspergillus tubingensis*	–
	62	SZMC 2040	*Aspergillus udagawae*	–
	63	SZMC 2041	*Aspergillus udagawae*	–
	64	SZMC 21694	*Aspergillus viridinutans*	–
	65	SZMC 13F	*Fusarium sacchari*	–
	66	SZMC 90/11	*Fusarium napiforme*	–
	67	SZMC 173/11	*Fusarium delphinoides*	–
	68	SZMC 394/11	*Fusarium oxisporum*	–
	69	SZMC FSU9682	*Lichtemia corymbifera*	–
	70	SZMC RH59	*Rhizopus oryzae*	43.13, 77.38
	71	SZMC Sce	*Scedosporium aurantiacum*	37.54, 75.73
Gram-positive bacteria	Reference	ATCC 25923	*Staphylococcus aureus*	–
	72	SZMC 14529	*Staphylococcus aureus*	–
	73	SZMC 14530	*Staphylococcus aureus*	–
	74	SZMC 14532	*Staphylococcus aureus*	–
	Reference	ATCC 29213	*Staphylococcus aureus*	–
	Reference	ATCC 43300	*Staphylococcus aureus*	–
	75	SZMC 14531	*Staphylococcus epidermidis*	–
	Reference	ATCC 29212	*Enterococcus faecalis*	–
	Reference	ATCC 8043	*Enterococcus hirae*	–
	76	SZMC 14538	*Enterococcus faecalis*	–
Gram-negative bacteria	Reference	ATCC 13048	*Enterobacter aerogenes*	–
	77	SZMC 6222E	*Enterobacter gergoviae*	–
	78	SZMC 6223	*Enterobacter gergoviae*	–
	79	SZMC 6224	*Enterobacter gergoviae*	–
	80	SZMC 21890	*Enterobacter cloacea*	–
	81	SZMC 21892	*Enterobacter cloacea*	–

Clinical isolates are from the Szeged Microbiology Collection (SZMC) and from the Debrecen Microbiology Collection (DMC). Reference strains are from American Stock Centre (ATCC), Centraalbureau voor Schimmelcultures, Fungal and Yeast Cultures (CBS), Northern Regional Research Laboratories (NRRL), Fungal Genetics Stock Centre (FGSC)

### DNA extraction

All genomic DNA (gDNA) extraction steps were performed in a class II laminar-flow cabinet to avoid environmental contamination.

#### Fungal gDNA extraction

*Candida* cultures used for the molecular identification of the strains were grown on yeast-peptone D-glucose (YPD) for 2 days, and DNA was extracted using the Masterpure™ Yeast DNA Purification Kit (Epicentre Biotechnol., Madison, USA) per the manufacturer’s instructions [42]. Mould reference strains (*Aspergillus* only) and clinical isolates (*Aspergillus*, *Fusarium*, *Lichtemia*, *Rhyzopus*, *Scedosporium*) were cultivated on standard minimal nitrate medium [43]. DNA extractions were carried out at the University of Debrecen (Department of Biotechnology and Microbiology). gDNA was isolated from liquid cultures grown in minimal medium at 37 °C (*A. fumigatus, A. niger*), 25 °C (*A. terreus*, *A. lentulus, A. flavus*) and 30 °C (*A. welwitschiae*) at 220 rpm for 18 h. The mycelium was disrupted by using Roche MagNa Lyser (Roche Diagnostics, Risch-Rotkreuz, Switzerland) and gDNA was isolated using the Genomic DNA Purification Kit (Thermo Scientific, Maryland, USA) per the manufacturer’s instructions.

#### Bacterial gDNA extraction

The bacterial strains were grown on Müller-Hinton agar base under aerobic conditions. DNA was extracted using the E.Z.N.A.® Bacterial DNA Kit (Omega Biotech, Norcross, Georgia, USA) per the manufacturer’s instructions. Briefly, for suspension cultures one millilitre of log phase culture with approximately 10^8^ bacterial cells was used and cells were pelleted by centrifugation. Gram-positive bacterial cell walls were lysed by lysozyme solution with 20 mg ml^− 1^ enzyme in 20 mM Tris HCL, pH 8.0 2 mM EDTA, 1.2% TritonX100).

DNA concentrations and purity were measured using NanoDrop-1000 spectrophotometer (NanoDrop Technologies, Inc., North Carolina, USA).

### CanTub-simplex PCR

Annotated sequences of the *Candida* beta-tubulin genes were extracted from the EMBL/GeneBank databases to make multiple alignments using Clustal Omega. Available *Candida* beta-tubulin sequences were aligned surveying potential sequence deviations within species. When designing primers, we screened for melting domains covering enough mismatches to enable proper discrimination among the tested strains but comprising 30–40 nucleotide long conserved nucleotides on flanking regions for the very specific hybridization of primers and avoiding cross-reactions with gDNA of other BSI causing fungal, bacterial strains and with the human genomic DNA.

The CanTub forward primer is 37 bp long(5’-CTAAAATCAGAGAAGAATTCCCCTGATAGAATGATGGC-3′), GC content 37.8%, Tm 73.2 °C and the CanTub reverse primer is 43 bp long (5’-CAATTGACCTGGGATAACGTAAAGAAGTAGTAACACCAGACAT-3′), GC content 38%, Tm 74.2 °C.

### Setting the optimal CanTub-simplex PCR conditions

Temperature gradient assay was performed from 55 to 72 °C for assessing the performance of the primer pair during amplification with a temperature gradient program using the LightCycler® 96 thermal cycler Instrument (Roche Applied Science, Penzberg, Germany). The MgCl_2_ optimization was performed by adding different amounts of MgCl_2_ within the concentration range 1 to 3.5 mM. The primer optimization assay was performed using 0.2, 0.4, 0.6 μM of the forward and reverse primers.

### Verification of the CanTub-simplex amplicons via conventional PCR

In order to monitor the accumulation of aspecific amplicons in the CanTub-simplex PCR, primers were tested with 20 ng gDNA of five *Candida* reference strains (*C. albicans* ATCC 10231, *C. glabrata* ATCC 90030, *C. parapsilosis* ATCC 22019, *C. tropicalis* ATCC 750, *C. krusei* ATCC 6258), and two clinical isolates (*C. guilliermondii* SZMC 1536 and *C. dubliniensis* SZMC 1470 depicted by ID33 and ID36 in Table 1). The thermocycling reactions were conducted in a Roche LightCycler®Nano instrument (P2). 200 ng of the yielded CanTub PCR amplicons were electrophoresed on 2% TBE-agarose gel stained with ethidium-bromide to investigate aspecific PCR increments.

### Real time PCR platforms

Real-time PCR assays were conducted on three different PCR platforms (P1, P2, P3). (P1) LightCycler® 96 thermal cycler, (P2) LightCycler® Nano and (P3) LightCycler® 2.0 Instruments (Roche Diagnostics, Risch-Rotkreuz, Switzerland) were used with the LightCycler 480 High-Resolution Melting Master (Roche Applied Science, Penzberg, Germany) **(P1)** 10 μl reaction volumes consisted of 5 μl 2× LightCycler 480 High Resolution Melting Master, 0.5 μl of each primer (0.4 μM), 1 μl MgCl_2_ (2 mM) and 3 μl template DNA. The thermocycling reactions were conducted in a LightCycler 480 Multiwell Plate, (white). **(P2), (P3)** 20 μl reaction volumes consisted of 10 μl 2× LightCycler 480 High Resolution Melting Master, 0.5 μl of each primer (0.4 μM), 5 μl PCR grade water, 1 μl MgCl_2_ (2 mM) and 3 μl template DNA. The thermocycling reactions were conducted in LightCycler® 8-Tube Strips (P2) and in 20 μl glass capillaries (P3). The real-time PCR runs always included at least two controls of reaction mix without DNA (non-template control - NTC). Temperature parameters were set as follows: an initial denaturing step of 95 °C for 10 min followed by 50 cycles of denaturation at 95 °C for 10 s, annealing at 60 °C for 10 s and extension at 72 °C for 10 s. Fluorescent data were collected in the ResolightDye channel (470/514 nm).

#### Melting temperature calculation and HRM protocols

The accumulation of the *Candida* species descriptive amplicons was monitored via melting temperature calculation and HRM analysis on three real-time PCR platforms.

**(P1), (P2)** Following the completion of real-time PCR, the products were denatured at 95 °C for 15 s (4.4 °C s^− 1^), and then renatured at 40 °C for 15 s (1 °C s^− 1^) to form DNA duplexes. HRM analysis was performed by increasing the temperatures from 45 to 97 °C (0.2 °C s^− 1^) recording changes in fluorescence (-dF/dT) and plotting against changes in temperature. The HRM profiles were then analyzed using the LightCycler®96 HRM analysis and the LightCycler® Nano Software thus highly dense measurement points were taken during the high resolution melting stage resulting in species descriptive HRM melting curves.

**(P3)** HRM analysis was not performed with the LC 2.0 software due to the lack of this option. For T_m_ analysis melting temperature measurement was set from 45 to 97 °C (0.05 °C s^− 1^). Melting peaks were analyzed by the LightCycler®2.0 Software.

### Cross reactivity

Possible cross reactions of the CanTub-HRM assay were tested with approximately 20–25 ng purified (OD_260/280_1.82–1.97) gDNA of human placenta (Sigma Aldrich, Missouri, USA) and the gDNA samples of 32 aspergilli; 6 reference strains (*Aspergillus terreus* FGSC A1156*, A. fumigatus* Af293, *A. lentulus* CBS117885, *A. flavus* NRRL11611, *A. niger* CBS 113.46, *A. tubingensis* CBS 134.48), further 26 gDNA of *Aspergillus* (ID39–64), *Fusarium* (ID65–68), *Lichtemia corymbifera* (ID69), *Rhyzopus oryzae* (ID70), *Scedosporium aurantiacum* (ID71) clinical isolates, finally with the gDNA of 10 Gram-positive bacteria; 5 reference strains (*Staphylococcus aureus* ATCC 25923, 29,213, 43,300, *Enterococcus faecalis* ATCC 29212, *Enterococcus hirae* ATCC 8043), 5 Gram-positive clinical isolates (ID72–76) and with 6 Gram-negative bacteria; *Enterobacter aerogenes* ATCC13048 reference strain and five Gram-negative clinical isolates (ID77–81) in Table 1.

### Verification of the CanTub-simplex assay on EDTA-whole blood panels

#### Obtaining of EDTA-WB samples for spike in controls

Working with WB samples from healthy volunteers was approved by the local ethics committee, MK-JA/50/0096–01/2017. Participants agreed to take part in this study. EDTA whole blood samples obtained from healthy volunteers were pooled and screened for *Candida* contamination prior to use as extraction negative controls (ENC).

#### *Candida* EDTA-WB reference panels

To obtain *Candida* reference panels in a 6-log range (*Candida albicans* ATCC 10231; ref-panel_1, *C. glabrata* ATCC 90030; ref-panel_2, *C. parapsilosis* ATCC 22019; ref-panel_3, *C. tropicalis* ATCC 750; ref-panel_4, *C. krusei* ATCC 6258; ref-panel_5, *C. guilliermondii* SZMC1536; ref-panel_6, *C. dubliniensis* SZMC 1470; ref-panel_7), 990 μl of EDTA-WB samples were seeded with 10 μl of log-phase culture suspensions at a cell density of 10^8^–10^2^ CFU in one millilitre of yeast suspensions. Following DNA purification the sample extracts contained 2 × 10^5^ (10^6^ CFU/1 ml WB) – 0.2 (10 CFU/1 ml WB) GE in 3 μl of PCR template.

#### *Candida* EDTA-WB clinical panels

EDTA-WB samples at a cell density of 10^4^ CFU/ml *Candida* strains (ID1–38) were used to perform seven *Candida* clinical panels (ID1–7; clin-panel_1, ID8–11; clin-panel_2, ID12–17; clin-panel_3, ID18–24; clin-panel_4, ID25–30; clin-panel_5, ID31–34; clin-panel_6, ID35–38; clin-panel_7). Following DNA purification the sample extracts contained 200 GE in 3 μl of PCR template.

#### DNA purification from EDTA-WB samples

DNA purification steps were performed in a class II laminar air-flow cabinet to avoid environmental contamination. Spiked EDTA-WB samples were disrupted (2000 rpm, 1 min) by Roche MagNa Lyser (Roche Diagnostics, Risch-Rotkreuz, Switzerland), and DNA was extracted along with the ENCs using the High Pure Viral Nucleic Acid Large Volume Kit (Roche Applied Science) according to manufacturer’s instructions. To obtain technical duplicates every sample extractions were performed in parallel. The elution volumes were adjusted to 15 μl and pooled.

### PCR efficiency and the limit of reliable detection (LoRD)

Amplification reactions were carried out on three different PCR platforms; P1, P2, P3. Duplicate PCRs were performed at every dilution of the *Candida* EDTA-WB reference panel extracts. Quantification cycle (Cq) values were subtracted and melting curves were analyzed to estimate the lowest template DNA concentration by which the appropriate, species descriptive melting peaks were interpretable (LoRD). To study the correlation between the Cq-s and the genomic load standard curves were obtained by plotting Cq values against the log of cell number; 10^6^–10 CFU/1 ml WB which is equivalent to 2 × 10^5^–0.2 GE/PCR. Mean Cq data were calculated and standard curves were built where these plots determined the linear dynamic ranges. Efficiency was calculated per the following formula, E = (10–1/slope) than was converted to percentage efficiency by using the formula, E% = (E-1) × 100 [44]. Amplification efficiency, (correlation coefficient; R^2^) was also calculated.

### In-house quality assessment

PCRs were conducted on P1 and T_m_ data were subtracted.

#### Determining the repeatability

To estimate repeatability intra-assay consistency was calculated. To calculate the coefficient of variation (% C.V.) of the CanTub-simplex PCR within the three PCR plates (ref-plate_1, _2, _3) of the seven *Candida* reference panel extracts (ref-panel_1-_7) and within the four PCR plates (clin-plate_1, _2, _3, _4) of the seven *Candida* clinical panel extracts (clin-panel_1-_7) standard deviation (±SD) of duplicate T_m_ mean data were taken, dividing these numbers by the mean of the T_m_ values and multiplying them by 100 [see Additional file 1]. Finally, the grand mean of the sample coefficient of variations (average % C.V.-s) of the three plates was taken defining the plate % C.V. values. When the intra-assay % C.V. is below 10% the method under investigation has high precision.

#### Determining the reproducibility

To estimate the precision of the CanTub-simplex PCR as regards discrimination of the relevant *Candida* species and to confirm that results generated are consistent over time inter-assay consistency (plate-to-plate variation) was estimated between the PCR plates on three distinct days with the seven *Candida* reference panel extracts and with the sample extracts of the seven *Candida* clinical panels [see Additional file 2]. In the case of the *Candida* panels duplicate PCRs were performed on every sample isolate. T_m_ data were subtracted than mean T_m_ (°C) with standard deviations (±SD) were calculated. When analyzing *Candida* clinical panels, duplicate T_*m*_ means of adherent clinical strains of the different species were assembled and overall mean was calculated. Plate coefficient of variations (% C.V.) was calculated. Finally, grand mean of the sample coefficient of variations (average % C.V.-s) was taken. Inter-assay % C.V. values less than 15% are generally acceptable.

## Results

### In silico assessment of the CanTub forward and CanTub reverse primers

Based on the sequence alignment data we presumed that the amplicons generated by CanTub-simplex primers will display enough sequence divergence between closely related *Candida* species but at the same time may be conserved enough barcoding the different clinical isolates within species. Primer matches with annotated beta-tubulin genes from *C. albicans*, *C. tropicalis*, *C. dubliniensis* and *C. glabrata* are indicated in [see Additional file 3] with the sizes of the amplicons.

### Optimal reaction conditions with the CanTub-simplex PCR

Optimal reaction conditions were determined as described in the methods section. 0.4 μM primers-, 2 mM MgCl_2_-concentrations and annealing at 60 °C proved to be optimal. The size of the amplified CanTub-simplex PCR bands were verified on five *Candida* reference strains and on two clinical isolates. All the amplified CanTub-simplex PCR fragments are smaller than 300 bp in size and we did not experience any aspecific PCR increments (data not shown).

### Cross reactivity

No cross-amplification was detected with the human genomic DNA and with the Gram-negative and Gram-positive bacteria. Furthermore, no cross-amplification was observed with *A. terreus, A. lentulus, A. tubingensis, A. viridinutans* and *A. udagawae* strains*.* Mild cross reaction was detected with the *A. fumigatus, A. flavus* and *A. niger* providing T_m_ peaks at 86.35 ± 0.16 °C and displaying inconclusive HRM curves and peaks. Moderate false positivity with plain, inconsistent peaks were detected in the case of *Rhyzopus oryzae* (77.38 °C), *Scedosporium aurantiacum* (75.73 °C) generating cycle threshold (Cq) values greater than Cq > 42 (data not shown).

### Analytical sensitivity

**Fig. 1:**
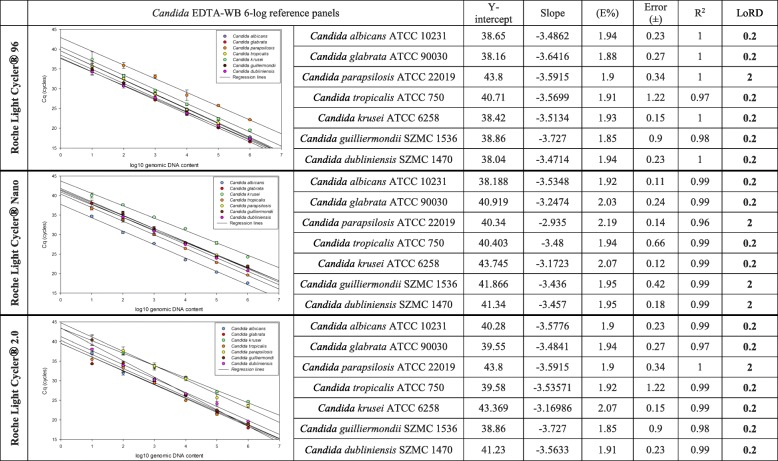
Regression lines of CanTub-simplex PCR on P1-P3 platforms. The limit of reliable detection (LoRD) was evaluated on seven, purified *Candida* EDTA-WB reference panels of the *Candida albicans* (ATCC 10231), *Candida glabrata* (ATCC 90030), *Candida parapsilosis* (ATCC 22019), *Candida tropicalis* (ATCC 750), *Candida krusei* (ATCC 6258) type strains and the *Candida guilliermondii* (SZMC 1536) and *Candida dubliniensis* (SZMC 1470) clinical isolates. Y-intercept, slope, PCR reaction efficiency (E%), error (±), coefficient of correlation (R^2^), and limit of reliable detection (LoD) measured in 20 μl PCR reactions are shown

LoRD of the simplex CanTub-PCR assay was estimated on *Candida* EDTA-WB standard panel extracts containing serially diluted *Candida* gDNA in a 6-log range. Linear dynamic ranges were assessed on P1, P2, and P3 with PCR efficiency (E) and coefficient of correlation (R^2^) were also estimated using the standard curve data (Fig. 1). The analytical sensitivity with the lowest concentration of template DNA where reliable identifications with conclusive melting peaks and HRM curves were attainable in the case of at least one technical duplicate proved to be 0.2 GE/PCR (10 cells/1 ml WB) on all panels and on all platforms except the *C. parapsilosis* reference panel on P1 and P3, where LoRD proved to be 2 GE/PCR (100 cells/1 ml WB) only. On P2 the LoRD was 2 GE/PCR also for *C. parapsilosis*, *C. dubliniensis* and *C. guilliermondii* reference panels.

### Maximum discriminatory property was achieved by defining seven melting clusters on three real-time platforms

CanTub-simplex assay targeting the beta-tubulin genes proved to be accurate and reliable for specific identification of seven *Candida* species. Thirty eight clinical isolates phenotypically identified as *Candida* species were subsequently tested in a blinded manner and barcoded due to comparing the melting data to those of the reference strains.

**Fig. 2:**
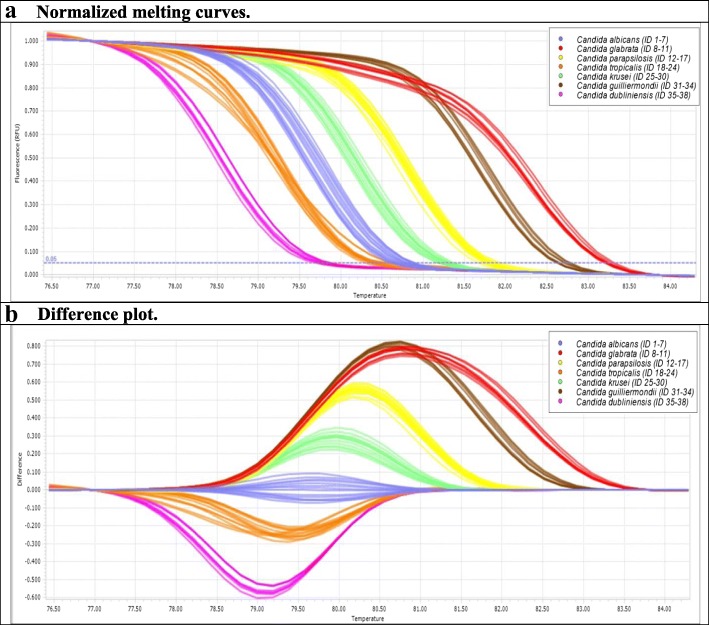
Discriminatory property of the CanTub-simplex PCR. Species level identification was achieved by using CanTub-simplex PCR primer pair to accurately identify *Candida* species based on HRM analysis (P1) by using the LightCycler®96 HRM analysis Software (Roche Diagnostics, Risch-Rotkreuz, Switzerland). **a** Representation of the normalized CanTub-simplex PCR melting curves. Each colour represents a different species: *Candida albicans* (blue) ID1–7; *Candida glabrata* (red) ID8–11; *Candida parapsilosis* (yellow) ID12–17; *Candida tropicalis* (orange) ID18–24; *Candida krusei* (green) ID25–30; *Candida guilliermondii* (brown) ID31–34; *Candida dubliniensis* (pink) ID35–38. **b** Representation of the normalized difference plots. Normalized difference plots against *Candida albicans*

Normalized melting curves (Fig. 2/a) with the difference plot (Fig. 2/b) are shown by using saturating concentrations of a fluorescent double stranded DNA intercalating ResoLight® dye. HRM analyses were performed on *Candida* EDTA-WB clinical panels and on platform P1.

**Fig. 3:**
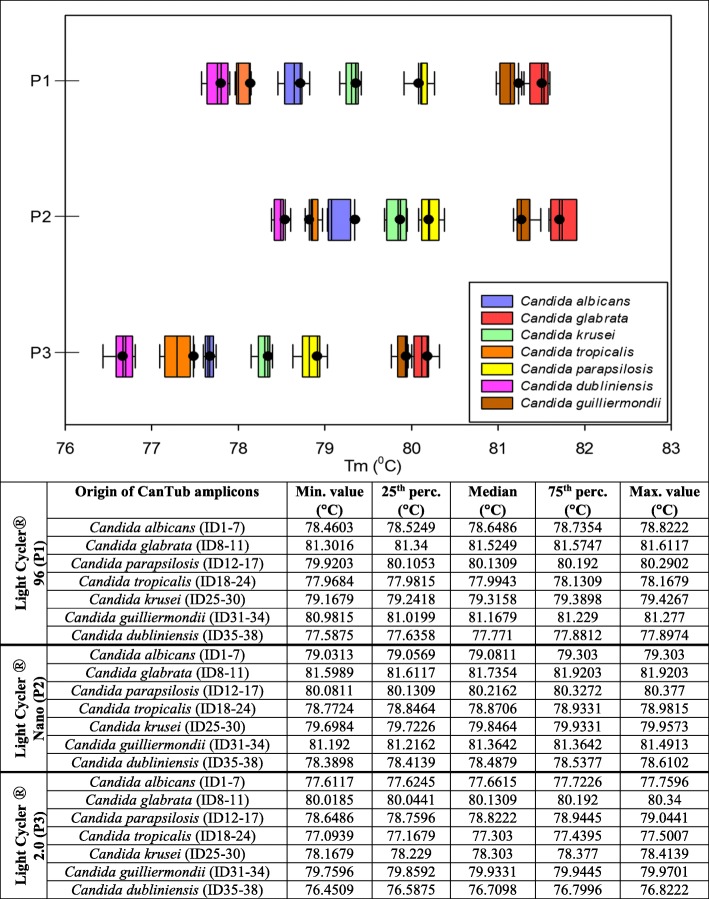
Whisker plots of the *Candida* species descriptive clusters. Whisker plots representing the discriminatory power of the CanTub-simplex PCR were created based on the digitalized melting temperature (T_m_) data representing the seven melting clusters (pink cluster_1 for *Candida dubliniensis*, orange cluster_2 for *Candida tropicalis*, blue cluster_3 for *Candida albicans*, green cluster_4 for *Candida krusei*, yellow cluster_5 for *Candida parapsilosis*, brown cluster_6 for *Candida guilliermondii*, red cluster_7 for *Candida glabrata*) for the discrimination of the *Candida* species. Melting peaks were subtracted from the analysis of purified the *Candida* EDTA-WB clinical panel extracts (P1-P3). T_*m*_ data of adherent clinical strains of the different species were assembled and data were grouped. Whisker plots were constructed for each data group showing the range of obtained temperatures of melting (T_m_); the minimum, the median, and the maximum T_m_ values with 25th and 75th percentiles

Whisker plots show the distribution of the T_m_ values when testing the 38 *Candida* clinical strains on four separate plates. For every dataset, the median, minimum, maximum T_m_ values along with the 25th and 75th percentile are also shown in Fig. 3. Based on the distribution of the melting temperature data of the *Candida* strains tested, we created seven melting clusters for the reliable species level identification on P1-P3 *C. dubliniensis* **cluster_1** T_m_ with 95% CI: 77.58 ± 0.32 °C (P1), 78.39 ± 0.22 °C (P2), 76.45 ± 0.37 °C (P3). *C. tropicalis* **cluster_2** T_m_ with 95% CI: 77.96 ± 0.2 °C (P1), 78.77 ± 0.21 °C (P2), 77.09 ± 0.41 °C (P3). *C. albicans* **cluster_3** T_m_ with 95% CI: 78.64 ± 0.18 °C (P1), 79.16 ± 0.14 °C (P2), 77.61 ± 0.15 °C (P3). *C. krusei* **cluster_4** T_m_ with 95% CI: 79.17 ± 0.26 °C (P1), 78.64 ± 0.18 °C (P2), 78.17 ± 0.24 °C (P3). *C. parapsilosis* **cluster_5** T_m_ with 95% CI: with 95% CI: 79.92 ± 0.37 °C (P1), 80.08 ± 0.3 °C (P2), 78.65 ± 0.39 °C (P3). *C. guilliermondii* **cluster_6** T_m_ with 95% CI: 80.98 ± 0.3 °C (P1), 81.19 ± 0.3 °C (P2), 79.76 ± 0.18 °C (P3). *C. glabrata* **cluster_7** T_m_ with 95% CI: 81.3 ± 0.31 °C (P1), 81.6 ± 0.32 °C (P2), 82.02 ± 0.32 °C (P3).Using these melting clusters for PCR typifying of unknown *Candida* strains there is no need for using standards as reference controls.

### Repeatability and reproducibility

We estimated the intra-, and inter-assay consistency of the CanTub-simplex PCR on *Candida* EDTA-WB reference- [see Additional file 1] and on *Candida* EDTA-WB clinical panels [see Additional file 2]. For measuring the precision of the assay average coefficient of variation was calculated for the technical duplicate T_m_ values measured on P1 where intra-assay % C. V. proved to be 0.14 (ref. plate_1), 0.19 (ref. plate_2) and 0.08 (ref. plate_3) on *Candida* EDTA-WB reference plates and 0.14 (clin. plate_1), 0.12 (clin. plate_2), 0.10 (clin. plate_3), 0.13 (clin. plate_4) on *Candida* EDTA-WB clinical plates accounting for a very high accuracy of the CanTub-simplex PCR. Plate-to-plate consistency was also assessed for the assay between the three *Candida* EDTA-WB reference plates and the four *Candida* EDTA-WB clinical panels. Reproducibility was measured and sample coefficient of variations (grand % C.V.-s) between the three reference and four clinical plates were taken, where inter-assay proved to be 0.11between standard [see Additional file 1] and 0.12between clinical panels [see Additional file 2] which is highly acceptable.

## Discussion

With the continuously growing population of immunocompromised patients, the number of invasive fungal infections has increased significantly over the past decades [1–4]. Among all, *Candida* species are the most prevalent etiology of sepsis and septic shock in critically ill patient groups representing a significant health challenge with increasing medical and economic importance [7–9]. Concern is rising about the growing incidence of non-*albicans* infections and the emergence of their various intrinsic or acquired resistances. Over the last two decades, the occurrence of non-*albicans* species have emerged [12, 13, 17, 18, 26]. More than 98% of candidemia cases are caused by *C. albicans*, *C. glabrata*, *C. parapsilosis*, *C. tropicalis*, *C. krusei*, including further species such as *C. dubliniensis,* and *C. guillermondii* [14–16].

Identification of *Candida* species is important due to the differences in the antifungal susceptibility profile associated with species and because of the limitation of the phenotypic identification [10–17].

The traditional culture based morphological (colony and microscopic morphology), and/or biochemical (such as sugar assimilation and fermentation tests) identification methods of the *Candida* species are laborious, requiring a high level of skills and expertise of clinical mycologists and a long period of time, limiting patient care [27, 28]. Morphological identification of certain species remains problematic due to the high degree of phenotypic similarities between *C. albicans* versus *C. tropicalis* and *C. dubliniensis* out of which the latter species can acquire stable fluconazole resistance rapidly [28].

There is an increasing demand for innovative sensitive, rapid and non-invasive methods for identification of *Candida* species at an early stage of the disease.

T_m_ calling assays especially when coupled with HRM analysis has been introduced for scanning genotypes and for the rapid discrimination between DNA sequences based on their variant high resolution melting profiles without the use of fluorescent labeled probes. Although HRM based methods do not have the resolving power that many sequencing techniques have and are not as sensitive as the TaqMan probe based systems, they became more and more attractive to molecular diagnostic laboratories. Our recent data also support that applications resting on HRM analyses may be ideally suited for identifying or ruling out certain fungal and bacterial pathogens in clinical diagnostic workflow.

In 2007 Carvalho and colleagues developed and tested on 231 *Candida* isolates a sensitive (2.15 ± 0.25 CFU/1 ml), multiplex PCR based method by using yeast specific (ITS1, ITS2) universal and species-specific (18S, 28S) primers allowing the barcoding of eight relevant *Candida* strains (*C. albicans, C. glabrata, C. parapsilosis, C. tropicalis, C. krusei, C. guilliermondii, C. lusitaniae and C. dubliniensis*) at the species level taking the advantage of the presence of high-copy number of rRNA genes due to the presence of characteristic PCR amplified band patterns [36]. The only drawback of this method is that barcoding of unknown yeasts require running eight simultaneous PCR assays.

The CanTub-simplex PCR assay described here relies on a single primer pair targeting specific regions of *Candida* beta-tubulin genes.

The assay provides reliable nucleic acid based testing for proper identification of seven relevant *Candida* species by defining the species-specific melting domains and/or the shape of the derivative melting curves on the three real-time platforms (P1-P3).

When analyzing 1 ml of WB samples infected with 10^6^–10 *Candida* cells the amplification efficiency was 100% on all reference panels (ref. panel_1 – ref. panel_7) observing efficient amplification and melt curve analysis. For samples seeded with 1 CFU sample the barcoding capacity proved to be only 78.57%. Nevertheless, on all *Candida* reference panels the amplification efficiencies were reliable (E% = 1.85–1.94). It must be also noted, that the standard deviations (mean SD: ±0.12 °C) of the T_m_ data measured on reference panels proved to be low even in bright concentration ranges.

Here we demonstrated that when appropriate DNA samples are available (OD_260/280_1.82–1.97 and gDNA concentration of 1–10 ng/μl), CanTub-simplex PCR assay identifies the seven most frequent pathogenic *Candida* species. CanTub-simplex PCR based applications used in parallel with morphotyping may offer better resolution of the species level identification.

This method is technically not demanding and provides clear, easily traceable protocols on three different real-time platforms. Single locus primer pair is used so the method can be easily multiplexed with exogenous or endogenous TaqMan internal control assays. Here we also demonstrated that the adaptation of DNA based applications used in tandem with morphological examinations in clinical diagnostic laboratories can offer better resolution of species within the genus.

## Conclusion

CanTub-simplex PCR targeting the beta-tubulin genes proved to be sensitive and accurate on all platforms (P1 – P3) tested here, and has the potential to identify seven clinically relevant *Candida* species (*C. albicans, C. glabrata, C. parapsilosis, C. tropicalis, C. krusei, C. guilliermondii, C. dubliniensis*) by using the seven, pre-defined melting clusters without using reference controls. These features make this CanTub-simplex PCR advantageous for use as a first-pass diagnostic adjunct in microbiology laboratories on blood culture bottles or for direct whole blood testing by making the patient follow-up more achievable.

## Additional files

Additional file 1: **Table S1.** Intra-, and inter-assay coefficient of variation of the CanTub-simplex PCR on *Candida* reference panels. Representation of the technical duplicates of the T_m_ data with mean and ± SD measured on *Candida* EDTA-WB reference panels at every dilution in a 6-log range. Intra-assay coefficient of variation was estimated for the particular gDNA panels on separarte plates (% C.V.-s of *Candida* ref-panel_1–7) even as for the plates in a whole (% C.V.-s of ref-plate_1–3). Inter-assay coefficient of variation was estimated for the CanTub-simplex PCR for the reference samples of all panels at every dilution in a 6-log range between three plates finally, grand mean of the sample coefficient of variation (grand % C.V. on ref-plates) was calculated. (DOCX 37 kb)

Additional file 2: **Table S2.** Intra-assay coefficient of variation of the CanTub-simplex PCR on 38 *Candida* clinical strains via EDTA-WB *Candida* clinical panel. T_m_ data of *Candida* clinical strains were grouped according to panels (clin-panel_1–7) and T_m_ mean was calculated with ±SD. Coefficient of variation (% C.V.) was calculated for every species. Finally, intra- and inter-assay consistencies were calculated. (DOCX 21 kb)

Additional file 3: **Figure S3.** CanTub HRM primer alignment results using BLAST algorithm. a) Primer positioning in the *Candida albicans* beta-tubulin gene with an amplicon size of 203 basepairs. b) Primer positioning in the *Candida dubliniensis* beta-tubulin gene with an amplicon size of 202 basepairs. c) Primer positioning in the *Candida tropicalis* beta-tubulin gene with an amplicon size of 204 basepairs. d) Primer positioning in the *Candida glabrata* chromosome K with an amplicon size of 204 basepairs. (DOCX 38 kb)

## Abbreviations

% C.V: Coefficient of variation
±SD: Standard deviation
ATCC: American type culture collection
BDG: 1,3-ß-D-glucan
bp: Base pairs
BSI: Bloodstream infections
CanTub: *Candida* tubulin
CBS: Centraalbureau voor Schimmelcultures
CFU: Colony forming unit
Cq: Quantification cycle
E: Efficiency
EDTA: Ethylenediaminetetraacetic acid
ENC: Extraction negative controls
gDNA: Genomic DNA
GE: Genomic equivalent
HRM: High resolution melting
IC: Invasive candidiasis
ICU: Intensive care unit
ITS: Internal transcribed spacer
LoRD: Limit of reliable detection
MALDI-TOF: Matrix assisted laser desorption/ionization time-of-flight
MS: Mass spectrometry
NRRL: Agricultural research service culture collection
NTC: Non-template control
OD: Optical density
PCR: Polymerase chain reaction
rDNA: Ribosomal DNA
SZMC: Szeged microbiology collection
TBE: Tris/Borate/EDTA
Tm: Melting temperature
WB: Whole blood
YPD: Yeast-pepton-D-glucose
