# Supplementary material for: Validation of a simplex PCR assay enabling reliable identification of clinically relevant Candida species
Source: BMC Infect Dis. 2018 Aug 13;18:393. doi: 10.1186/s12879-018-3283-6 (PMC6090785; doi:10.1186/s12879-018-3283-6)
Supplement: Supplementary file 1 — Table S1. Intra-, and inter-assay coefficient of variation of the CanTub-simplex PCR on Candida reference panels. Representation of the technical duplicates of the Tm data with mean and ± SD measured on Candida EDTA-WB reference panels at every dilution in a 6-log range. Intra-assay coefficient of variation was estimated for the particular gDNA panels on separarte plates (% C.V.-s of Candida ref-panel_1–7) even as for the plates in a whole (% C.V.-s of ref-plate_1–3). Inter-assay coefficient of variation was estimated for the CanTub-simplex PCR for the reference samples of all panels at every dilution in a 6-log range between three plates finally, grand mean of the sample coefficient of variation (grand % C.V. on ref-plates) was calculated. (DOCX 37 kb) [file 12879_2018_3283_MOESM1_ESM.docx]

| Table S1. Intra-, and inter-assay coefficient of variation of the CanTub-simplex PCR on *Candida* reference panels. | | | | | | |
| --- | --- | --- | --- | --- | --- | --- |
|  |  | Intra-assay consistency | | | Inter-assay consistency | |
| Panels | gDNA/  PCR | ref-plate_1  Tm1 (°C) | ref-plate _2  Tm2 (°C) | ref-plate _3  Tm3 (°C) | T_m_ mean  ±SD | % C.V. |
| *Candida albicans*  (ATCC10231)  ref-panel_1 | 7.5 ng/2x10^5^ GE | 78.49, 78.51 | 78.71, 78.57 | 78.68, 78.50 | 78.57±0.09 | 0.11 |
|  | 0.75 ng/2x10^4^ GE | 78.71, 78.77 | 78.82, 78.76 | 78.70, 78.71 | 78.75±0.05 | 0.06 |
|  | 75 pg/2x10^3^ GE | 78.64, 78.68 | 78.74, 78.69 | 78.71, 78.82 | 78.71±0.06 | 0.08 |
|  | 7.5 pg/2x10^2^ GE | 78.73, 78.85 | 78.95, 78.84 | 78.74, 78.68 | 78.79±0.1 | 0.13 |
|  | 0.75 pg/20 GE | 78.92, 78.94 | 78.94, 78.88 | 78.66, 78.59 | 78.82±0.16 | 0.2 |
|  | 75 fg/2 GE | 78.77, - | 78.93, - | 78.76, - | 78.82±0.1 | 0.13 |
|  | T_m_ mean±SD | 78.74±0.14 | 78.80±0.12 | 78.70±0.1 |  | |
|  | panel_1 % C.V. | 0.2 | 0.15 | 0.13 |  |  |
| *Candida glabrata*  (ATCC90030) ref-panel_2 | 7.5 ng | 81.42, 81.46 | 81.43, 81.47 | 81.42, 81.44 | 81.44±0.02 | 0.02 |
|  | 0.75 ng | 81.38, 81.41 | 81.41, 81.36 | 81.43, 81.44 | 81.41±0.03 | 0.04 |
|  | 75 pg | 81.55, 81.62 | 81.42, 81.48 | 81.50, 81.48 | 81.51±0.07 | 0.09 |
|  | 7.5 pg | 81.48, 81.57 | 81.58, 81.55 | 81.46, 81.53 | 81.53±0.05 | 0.06 |
|  | 0.75 pg | 81.39, 81.44 | 81.66, 81.63 | 81.54, 81.59 | 81.54±0.11 | 0.13 |
|  | 75 fg | 81.57, 81.64 | 81.64, 81.58 | 81.62, 81.68 | 81.63±0.04 | 0.05 |
|  | T_m_ mean±SD | 81.49±0.09 | 81.52±0.10 | 81.51±0.08 |  | |
|  | panel_2 % C.V. | 0.11 | 0.12 | 0.01 |  |  |
| *Candida parapsilosis*  (ATCC22019) ref-panel_3 | 7.5 ng | 79.92, 79.91 | 79.89, 79.9 | 79.98, 80.12 | 79.95±0.09 | 0.11 |
|  | 0.75 ng | 80.12, 80.18 | 80.23, 80.17 | 80.25, 80.16 | 80.19±0.05 | 0.06 |
|  | 75 pg | 80.15, 80.09 | 79.96, 79.94 | 80.17, 80.15 | 80.08±0.1 | 0.12 |
|  | 7.5 pg | 80.11, 80.13 | 80.23, 80.15 | 80.23, 80.17 | 80.17±0.05 | 0.06 |
|  | 0.75 pg | 79.93, 79.96 | 80.12, 80.0 | 80.09, 80.07 | 80.03±0.08 | 0.1 |
|  | 75 fg | -, - | -, - | -, - | - | - |
|  | T_m_ mean±SD | 80.05±0.11 | 80.06±0.14 | 80.14±0.07 |  | |
|  | panel_3 % C.V. | 0.14 | 0.17 | 0.09 |  |  |
| *Candida tropicalis*  (ATCC 750)  ref-panel_4 | 7.5 ng | 78.15, 78.17 | 78.17, 78.16 | 78.29, 78.25 | 78.20±0.06 | 0.08 |
|  | 0.75 ng | 78.17, 78.13 | 78.27, 78.19 | 78.22, 78.28 | 78.21±0.06 | 0.08 |
|  | 75 pg | 78.03, 78.08 | 78.19, 78.13 | 78.27, 78.22 | 78.15±0.09 | 0.12 |
|  | 7.5 pg | 78.19, 78.07 | 78.2, 78.28 | 78.17, 78.29 | 78.2±0.08 | 0.10 |
|  | 0.75 pg | 78.28, 78.21 | 78.38, 78.39 | 78.20, 78.15 | 78.27±0.10 | 0.12 |
|  | 75 fg | 78.24, 78.15 | 78.22, 78.23 | 78.14, 78.27 | 78.21±0.05 | 0.06 |
|  | T_m_ mean±SD | 78.16±0.07 | 78.23±0.08 | 78.23±0.05 |  | |
|  | panel_4 % C.V. | 0.09 | 0.10 | 0.06 |  |  |
| *Candida*  *krusei*  (ATCC 6258)  ref-panel_5 | 7.5 ng | 79.26, 79.28 | 79.28, 79.27 | 79.4, 79.37 | 79.31±0.06 | 0.08 |
|  | 0.75 ng | 79.34, 79.39 | 79.28, 79.33 | 79.32, 79.28 | 79.32±0.04 | 0.05 |
|  | 75 pg | 79.45, 79.41 | 79.31, 79.39 | 79.45, 79.35 | 79.39±0.06 | 0.08 |
|  | 7.5 pg | 79.41, 79.48 | 79.5, 79.46 | 79.28, 79.33 | 79.41±0.09 | 0.11 |
|  | 0.75 pg | 79.29, 79.32 | 79.29, 79.3 | 79.34, 79.48 | 79.34±0.07 | 0.09 |
|  | 75 fg | 79.31, 79.36 | 79.55, 79.4 | 79.32, 79.29 | 79.37±0.1 | 0.13 |
|  | T_m_ mean±SD | 79.36±0.07 | 79.36±0.1 | 79.35±0.06 |  | |
|  | panel_5 % C.V. | 0.09 | 0.13 | 0.08 |  |  |
| *Candida guillermondii*  (SZMC 1536)  ref-panel_6 | 7.5 ng | 81.19, 81.03 | 81.02, 81.08 | 81.27, 81.24 | 81.14±0.11 | 0.14 |
|  | 0.75 ng | 81.23, 81.33 | 81.29, 81.28 | 81.02, 81.14 | 81.22±0.12 | 0.15 |
|  | 75 pg | 81.26, 81.34 | 81.32, 81.34 | 81.21, 81.33 | 81.30±0.05 | 0.06 |
|  | 7.5 pg | 81.21, 81.34 | 81.37, 81.34 | 81.28, 81.32 | 81.31±0.06 | 0.07 |
|  | 0.75 pg | 81.23, 81.28 | 81.24, 81.25 | 81.12, 81.17 | 81.22±0.06 | 0.07 |
|  | 75 fg | 81.11, 81.31 | 81.07, 81.16 | 81.42, 81.38 | 81.24±0.15 | 0.18 |
|  | T_m_ mean±SD | 81.24±0.1 | 81.23±0.12 | 81.24±0.12 |  | |
|  | panel_6 % C.V. | 0.12 | 0.15 | 0.14 |  |  |
| *Candida dubliniensis*  (SZMC 1470)  ref-panel_7 | 7.5 ng | 77.86, 77.67 | 77.67, 77.73 | 77.89, 77.90 | 77.79±0.11 | 0.14 |
|  | 0.75 ng | 77.88, 77.99 | 77.94, 77.93 | 78.03, 77.95 | 77.95±0.05 | 0.06 |
|  | 75 pg | 77.9, 78.05 | 78.0, 77.98 | 77.96, 77.93 | 77.97±0.05 | 0.06 |
|  | 7.5 pg | 77.89, 78.12 | 78.21, 78.07 | 77.92, 78.03 | 78.04±0.12 | 0.15 |
|  | 0.75 pg | 77.88, 77.91 | 78.09, 77.96 | 78.07, 77.98 | 78.15±0.47 | 0.6 |
|  | 75 fg | 77.9, 77.93 | 77.91, 77.94 | 77.89, 77.9 | 77.91±0.02 | 0.03 |
|  | T_m_ mean±SD | 77.92±0.11 | 77.95±0.15 | 77.95±0.06 | Grand % C.V. on  ref. plates: 0.11 | |
|  | panel_7 % C.V. | 0.14 | 0.19 | 0.08 |  |  |
|  | | Ref. plate_1  % C.V.: 0.13 | Ref. plate_2  % C.V.: 0.14 | Ref. plate_3  % C.V.: 0.08 |  | |

Representation of the technical duplicates of the T_m_ data with mean and ±SD measured on *Candida* EDTA-WB reference panels at every dilution in a 6-log range. Intra-assay coefficient of variation was estimated for the particular gDNA panels on separarte plates (% C.V.-s of *Candida* ref-panel_1-7) even as for the plates in a whole (% C.V.-s of ref-plate_1-3). Inter-assay coefficient of variation was estimated for the CanTub-simplex PCR for the reference samples of all panels at every dilution in a 6-log range between three plates finally, grand mean of the sample coefficient of variation (grand % C.V. on ref-plates) was calculated.
