# Supplementary material for: Validation of a simplex PCR assay enabling reliable identification of clinically relevant Candida species
Source: BMC Infect Dis. 2018 Aug 13;18:393. doi: 10.1186/s12879-018-3283-6 (PMC6090785; doi:10.1186/s12879-018-3283-6)
Supplement: Supplementary file 2 — Table S2. Intra-assay coefficient of variation of the CanTub-simplex PCR on 38 Candida clinical strains via EDTA-WB Candida clinical panel. Tm data of Candida clinical strains were grouped according to panels (clin-panel_1–7) and Tm mean was calculated with ±SD. Coefficient of variation (% C.V.) was calculated for every species. Finally, intra- and inter-assay consistencies were calculated. (DOCX 21 kb) [file 12879_2018_3283_MOESM2_ESM.docx]

**Table S2.** Intra-assay coefficient of variation of the CanTub-simplex PCR on 38 *Candida* clinical strains via EDTA-WB *Candida* clinical panel.

|  | clin-plate_1 | clin-plate_2 | clin-plate_3 | clin-plate_4 |
| --- | --- | --- | --- | --- |
|  | Mean of CanTub T_m_  (°C)±SD | Mean of CanTub T_m_  (°C)±SD | Mean of CanTub T_m_  (°C)±SD | Mean of CanTub T_m_  (°C)±SD |
| clin-panel_1  *Candida albicans*  (ID1-7) | 78.59±0.15 | 78.62±0.06 | 78.58±0.12 | 78.60±0.06 |
| % C.V. | 0.19 | 0.08 | 0.15 | 0.08 |
| clin-panel_2  *Candida glabrata*  (ID8-11) | 81.53±0.11 | 81.47±0.11 | 81.55±0.12 | 81.50±0.11 |
| % C.V. | 0.13 | 0.14 | 0.15 | 0.14 |
| clin-panel_3  *Candida parapsilosis*  (ID12-17) | 80.23±0.1 | 80.23±0.13 | 80.23±0.05 | 80.22±0.11 |
| % C.V. | 0.12 | 0.16 | 0.06 | 0.14 |
| clin-panel_4  *Candida tropicalis*  (ID18-24) | 78.03±0.09 | 78.04±0.08 | 77.99±0.1 | 78.02±0.10 |
| % C.V. | 0.12 | 0.1 | 0.13 | 0.13 |
| clin-panel_5  *Candida krusei*  (ID25-30) | 79.25±0.09 | 79.26±0.09 | 79.32±0.1 | 79.25±0.08 |
| % C.V. | 0.11 | 0.11 | 0.13 | 0.1 |
| clin-panel_6  *Candida guilliermondii*  (ID31-34) | 81.14±0.12 | 81.06±0.09 | 81.16±0.04 | 81.09±0.1 |
| % C.V. | 0.15 | 0.11 | 0.05 | 0.12 |
| clin-panel_7  *Candida dubliniensis*  (ID35-38) | 77.74±0.12 | 77.85±0.11 | 77.62±0.05 | 77.76±0.16 |
| % C.V. | 0.15 | 0.14 | 0.06 | 0.21 |
| Intra-assay consistency  Plate % C.V. | **0.14** | **0.12** | **0.10** | **0.13** |
| Inter-assay consistency  Grand % C.V. | **0.12** | | | |

T_m_ data of *Candida* clinical strains were grouped according to panels (clin-panel_1-7) and T_m_ mean was calculated with ±SD. Coefficient of variation (% C.V.) was calculated for every species. Finally, intra- and inter-assay consistencies were calculated .
