# Supplementary material for: Validation of a simplex PCR assay enabling reliable identification of clinically relevant Candida species
Source: BMC Infect Dis. 2018 Aug 13;18:393. doi: 10.1186/s12879-018-3283-6 (PMC6090785; doi:10.1186/s12879-018-3283-6)
Supplement: Supplementary file 3 — Figure S3. CanTub HRM primer alignment results using BLAST algorithm. a) Primer positioning in the Candida albicans beta-tubulin gene with an amplicon size of 203 basepairs. b) Primer positioning in the Candida dubliniensis beta-tubulin gene with an amplicon size of 202 basepairs. c) Primer positioning in the Candida tropicalis beta-tubulin gene with an amplicon size of 204 basepairs. d) Primer positioning in the Candida glabrata chromosome K with an amplicon size of 204 basepairs. (DOCX 38 kb) [file 12879_2018_3283_MOESM3_ESM.docx]

**b)**

**a)**

**d)**

**c)**

**Figure S3.** CanTub HRM primer alignment results using BLAST algorithm. **a)** Primer positioning in the *Candida albicans* beta-tubulin gene with an amplicon size of 203 basepairs. **b)** Primer positioning in the *Candida dubliniensis* beta-tubulin gene with an amplicon size of 202 basepairs. **c)** Primer positioning in the *Candida tropicalis* beta-tubulin gene with an amplicon size of 204 basepairs. **d)** Primer positioning in the *Candida glabrata* chromosome K with an amplicon size of 204 basepairs.
